# Editorial: Work–Life Balance: Essential or Ephemeral?

**DOI:** 10.3389/fped.2017.00108

**Published:** 2017-05-10

**Authors:** Andreas Schwingshackl, Kanwaljeet J. S. Anand

**Affiliations:** ^1^Pediatric Critical Care Medicine, University of California Los Angeles, Los Angeles, CA, USA; ^2^Pediatric Critical Care Medicine, Stanford University, Stanford, CA, USA

**Keywords:** work, life, balance, burnout, physician, academic medicine, stress, suicide

**Editorial on the Research Topic**

**Work–Life Balance: Essential or Ephemeral?**

An overwhelming response followed my inaugural article in *Frontiers in Pediatric Critical Care* in 2014 titled “*The fallacy of chasing after work–life balance*” (Schwingshackl). My article revealed the perspective of a young clinician-scientist trying to stay afloat between the responsibilities of a busy pediatric ICU service and a nascent basic science research career. While most professionals in academic medicine adhere to the notion that achieving a work–life balance is integral to the recipe for academic success, I challenged the usefulness and necessity of the work–life balance paradigm in modern medicine. I argued that creating a dichotomy between work and life leads down a self-destructive path filled with frustration and disappointment toward both “work” and “life.” In contrast, integration rather than separation of the time spent at work and with life may create a much more positive and constructive attitude toward both of these entities.

This provocative viewpoint sparked a remarkable debate and stirred up a plethora of emotions in MDs/DOs (Lin; Figueroa; Epstein; Kimura) and PhDs (You; Roan; Saravia and Saravia), including students, residents (Garros; Vargas; Fernandez Nievas and Thaver), fellows (Purdie; Alleyne), and faculty (Federman; Morparia; Shenoi), from all flavors of medicine and across the spectra of careers and life stages. Importantly, this is the first project that dissects this controversy not only by collecting the opinions of academic employees themselves but also the perceived views of their spouses and life partners (Saravia and Saravia) and in the context of mentor–mentee relationships (Fernandez Nievas and Thaver). Given the resonance of this topic in the academic community, this debate evolved into a Research Topic, allowing the expression of wide-ranging ideas, perspectives, personal testimonies, and coping strategies.

In this Research Topic, 26 authors contributed a total of 22 manuscripts, addressing for the first time the controversy of work–life balance from a 360° viewpoint. At its core, it highlights that all academic employees struggle with this concept in one way or another. During our careers, we have developed a wide range of coping strategies to deal with this issue. Some authors aim at resolving this conflict on a more small-scale and immediate, daily basis (Garros; Kong), whereas others adopt more long-term coping mechanisms, including frequent reassessments, and shy away from focusing too deeply on small ups and downs in a daily workday (Saini) Some authors prefer to digest this conflict first at philosophical and moral levels before implementing daily, practical strategies (Lin; Epstein). Others promote the concept that a strictly balanced work–life balance is unlikely achievable in our modern lives, whereas instead acceptance of a healthy work–life imbalance, in conjunction with a rearrangement of priorities, can realistically lead to satisfaction and happiness (Figueroa; Shenoi; Tarquinio). Along those lines, some authors point out that work–life balance for physician-scientists could be easier achievable if we rated ourselves on our own scale rather than those adopted from the business world, which inherently poorly reflect the nature of the conflicts that physicians struggle with (Fernandez Nievas and Thaver). Taking this controversy to another level, some authors remind us of the importance of realizing that work–life dissatisfaction not only manifests itself as an internal struggle but directly infiltrates our interactions with our families, friends, and others in our personal and professional circles (Kimura). In fact, it is well known that persistent work–life dissatisfaction can lead to burnout and depression among ICU physicians (1). Toward the extremes of the spectrum of possible approaches are authors who question the entire usefulness of the concept of work–life balance (You; Wang) or, like myself and others (Schwingshackl; Alleyne), reject the concept altogether and believe it is actually more harmful than useful to our serenity.

The ultimate goal of this Research Topic was to provide clinicians and clinician-scientists with a series of options of how to approach work–life balance in the world of modern academic medicine. Each manuscript shares personal experiences and commentaries on the pros and cons of a given approach and value system. Clearly, it is impossible to compile a one-approach-fits-all model for all clinicians. Nevertheless, each one of us can certainly relate to at least one of these approaches, recognize its benefits and downfalls, and gain new strength from adopting at least certain aspects of other portrayed strategies to fit our own specific needs.

Like many of us, we were inspired by the unprecedented increases in suicide and burnout rates among medical professionals (2–5), most likely caused by the gradual but consistent adaptation of characteristics and value systems typical for a business enterprise in modern medicine (6). Physician careers are increasingly driven by promotions and incentives (financial or otherwise) and are often viewed in light of achieving further promotions or access to additional perks or incentives (7–10). Furthermore, the increasing pressure of practicing cost-effective medicine, although essential for the sustainability of any health-care system, imposes ethical and moral stressors on physicians as it is often rewarded by further bonuses and incentives (11–14). Similarly, the implementation of business-derived productivity metrics to define a physician’s value to an academic institution is gradually replacing any human, emotional, and intellectual values that a physician brings to the workplace (15–19). We are being conditioned to suppress the ambitions and desires that originally led us into medicine and replace them with skill sets that allow us to sell ourselves as profitable investments. We write grants on topics with the highest likelihood of being funded, not on the topics closest to our hearts. We prefer seeing patients with fewer comorbidities, rather than intellectually challenging patients who pose a diagnostic dilemma, because of their impact on our productivity. Aside from adopting business-like practices in medicine, policy makers, accreditation bodies, and insurance companies have over the past two decades also imposed an unprecedented number of practice guidelines, documentation rules, and a mandate to implement one version or another of an electronic medical record (EMR) system. These have created a culture of fear of medical–legal lawsuits among physicians encouraging them to generalize and delegate rather than individualize and take ownership of a patient’s medical care. This in turn has negative effects on physicians’ self-esteem and makes them feel underappreciated. Hasty implementation of EMR systems promoted inner conflicts in many physicians due the contradiction between their individual perspective that virtually all EMR versions are inefficient and impractical (20), and questionable data proposing improved efficiency, quality of care, and financial outcomes (21, 22). Interestingly, some studies report not even any improvements over time in physicians’ satisfaction scores with EMR systems and point out serious deficiencies that hinder physicians’ routine work (23). These “advances” in medicine have clearly fueled dissatisfaction and frustration levels throughout the whole medical profession to an all-time high and have vastly changed our daily practice patterns.

An entirely new stressor imposed on our medical community, one that no other generation in the history of medicine had to cope with, is the fundamental instability of our health-care system. Introduction of the Affordable Health Care Act (“Obamacare”) in March 2010 certainly led to substantial anxiety among all practicing physicians about how it would affect our daily practice, workload, and personal lifestyle. However, not even 7 years later, our lives are upended once again with the transition to a new presidency that is promising not only to abolish the ACA but also, to date, has not proposed any alternative. We are currently facing higher than ever levels of anxiety and insecurity about the future of health care, the resources that allow us to practice standard-of-care medicine, and the ramifications on our compensation plans, all of which directly affect both our work and personal environments. No generation before ours had to face such a fundamental and existential uncertainty about the moral, ethical, and financial value of our profession and our personal value within the health-care system.

The recent shift in the mere definitions of both medicine and academic research suffocates our passions that originally inspired us to embark on the journey of helping sick children. These principles fuel inner conflicts that, in turn, we try to remedy with a work–life balance paradigm. Our Research Topic identified these conflicts as major stressors for modern clinician-scientists across all ages and life stages.

By now, the topic of work–life balance has clearly gained ample media coverage and public attention (24–28), which has increasingly led academic institutions to implement emotional support systems for physicians (29–32). Unfortunately, most of these approaches are once again copied from business corporation models and as long as resuscitating a sick child creates a completely different stressor profile than navigating through the ups and downs of the stock market, in reality these models provide little useful help for physicians. In fact, participating in such programs (or regular “MD wellness” surveys) often adds to our stress level rather than alleviating it. Widespread concerns also remain about the confidentiality and the stigma associated with mental health problems in physicians (33). The stigma of mental illness thrives in the medical profession as a result of the current health-care culture, the perceptions of physicians and their colleagues, as well as the regulatory burdens, expectations, and responses of health-care systems.

As a thought experiment, let us turn this model upside down and create a workplace where physician values are driven by purpose and initiative, both at work and in life. Once we recognize our purpose in both life and work, we can start defining the initiatives required to fulfill our inner purpose not only as physicians but also as human beings. Satisfaction and tranquility will then derive from living a purpose-driven life at work and outside with our coworkers, families, and friends alike, and not from financial enrichments or accumulation of titles on our business cards and email signatures. While we agree that we cannot just chase romantic ideals of making the world a better place, we cannot abandon those ideals either and practice medicine solely based on productivity metrics, promotion criteria, and cost-effectiveness analyses.

Personally, we challenge and refute the concept of work–life balance as a useful construct and consider it harmful to our search for happiness and physical and spiritual well-being. Nevertheless, this Research Topic has revealed that for the large majority of physicians, regardless of gender, age, academic rank, or size of institution, the concept of work–life balance is very much alive, constantly present in their daily realities and forces them to deal with its definition and ramifications every step of the way. It also provided a forum to express the wide-ranging perspectives, ideas, and strategies used to achieve work–life balance by professionals practicing in academic and non-academic medical settings. Encapsulating all these in a readily accessible electronic booklet will reach a wider audience and potentially help those struggling with the diverse stressors of our current medical climate.

Unquestionably, the landscape of modern academic medicine is changing. While we, the currently active physician work force, can opt to accept or decline the usefulness of a work–life balance concept to find our serenity, the next generation of clinician-scientists will likely have to redefine the question of “*Work–Life Balance: Essential or Ephemeral?*” for themselves based on their own future work–life contexts.

## Author Contributions

Both authors contributed equally to the planning and execution of this editorial.

## Conflict of Interest Statement

The authors declare that the research was conducted in the absence of any commercial or financial relationships that could be construed as a potential conflict of interest.
